# Therapeutic implications of *C. albicans-S. aureus* mixed biofilm in a murine subcutaneous catheter model of polymicrobial infection

**DOI:** 10.1080/21505594.2021.1894834

**Published:** 2021-03-08

**Authors:** Taissa Vila, Eric F. Kong, Daniel Montelongo-Jauregui, Patrick Van Dijck, Amol C. Shetty, Carrie McCracken, Vincent M. Bruno, Mary Ann Jabra-Rizk

**Affiliations:** a Department of Oncology and Diagnostic Sciences, School of Dentistry, University of Maryland, Baltimore, MD, USA; b Laboratory of Molecular Cell Biology, Institute of Botany and Microbiology, KU Leuven, Leuven-Heverlee, Belgium; c VIB-KU Leuven Center for Microbiology, Flanders, Belgium; d Institute for Genome Sciences, University of Maryland School of Medicine, Baltimore, MD, USA; e Department of Microbiology and Immunology, University of Maryland School of Medicine, Baltimore, MD, USA

**Keywords:** Candida, Staphylococcus, mixed-biofilms, polymicrobial infections, transcriptome

## Abstract

Biofilm-associated polymicrobial infections tend to be challenging to treat. *Candida albicans* and *Staphylococcus aureus* are leading pathogens due to their ability to form biofilms on medical devices. However, the therapeutic implications of their interactions in a host is largely unexplored. In this study, we used a mouse subcutaneous catheter model for *in vivo*-grown polymicrobial biofilms to validate our in vitro findings on *C. albicans*-mediated enhanced S. aureus tolerance to vancomycin *in vivo*. Comparative assessment of *S. aureus* recovery from catheters with single- or mixed-species infection demonstrated failure of vancomycin against *S. aureus* in mice with co-infected catheters. To provide some mechanistic insights, RNA-seq analysis was performed on catheter biofilms to delineate transcriptional modulations during polymicrobial infections. *C. albicans* induced the activation of the *S. aureus* biofilm formation network *via* down-regulation of the lrg operon, repressor of autolysis, and up-regulation of the ica operon and production of polysaccharide intercellular adhesin (PIA), indicating an increase in eDNA production, and extracellular polysaccharide matrix, respectively. Interestingly, virulence factors important for disseminated infections, and superantigen-like proteins were down-regulated during mixed-species infection, whereas capsular polysaccharide genes were up-regulated, signifying a strategy favoring survival, persistence and host immune evasion. *In vitro* follow-up experiments using DNA enzymatic digestion, lrg operon mutant strains, and confocal scanning microscopy confirmed the role of *C. albicans*-mediated enhanced eDNA production in mixed-biofilms on *S. aureus* tolerance to vancomycin. Combined, these findings provide mechanistic insights into the therapeutic implications of interspecies interactions, underscoring the need for novel strategies to overcome limitations of current therapies.

## Introduction

Polymicrobial infections caused by a combination of microorganisms are responsible for significant mortality and morbidity [1–3]. Biofilm-associated mixed infections, such as those on indwelling medical devices, are particularly challenging to treat due to their inherent heterogeneity, which can be further amplified by synergistic inter-species interactions [1,4–6]. Therefore, understanding the molecular inter-species interactions will greatly aid in designing novel therapeutic strategies targeting mixed biofilm infections, specifically those involving fungi and bacteria.

*Candida albicans* is the most common human fungal pathogen causing diseases ranging from superficial mucosal to life-threatening systemic infections [7–9]. The majority of *C. albicans* infections are associated with its ability to form biofilms where adhesion of yeast cells to the substrate is followed by proliferation and formation of hyphae, resulting in a network of cells embedded in an extracellular matrix [10–13]. *C. albicans* biofilm matrix is complex, with major polysaccharide constituents being α-mannan, β-1,6-glucan, and β-1,3-glucan which has been linked to resistance of biofilm to antifungals [14–17]. However, in various niches in the host, *C. albicans* coexists with numerous bacterial species, most notably *Staphylococcus aureus* a major human bacterial pathogen that causes a wide variety of diseases [18–24]. *S. aureus* alone is a poor former of biofilms, however, together with *C. albicans*, this species forms a substantial biofilm where the fungus creates a scaffold for the bacteria [6,25]. The growing use of implanted medical devices is another reason why the incidence of *Candida* and staphylococcal infections has steadily increased, since the majority of these infections are emerging from biofilms formed on medical implants [26,27].

Our previous characterization of mixed *C. albicans* and *S. aureus* biofilms *in vitro* and *in vivo* demonstrated that *S. aureus* exhibits high affinity to the *C. albicans* hyphal form as these species co-adhere and interact synergistically, forming a dense and architecturally complex biofilm [25,28,29]. Furthermore, using a mouse model of oral co-infection, we demonstrated that onset of oral candidiasis may lead to systemic staphylococcal infection, stemming from a thick matrix of mixed biofilm formed on tongue tissue [30,31]. Our subsequent *in vitro* studies into the therapeutic implication of *C. albicans-S. aureus* interactions in biofilm demonstrated that *C. albicans* secreted matrix polysaccharides, primarily β-1,3-glucan, confers *S. aureus* with enhanced tolerance to antimicrobials by impeding drug penetration through the biofilm [29]. However, findings from the study also indicated that other factors may also contribute to the enhanced *S. aureus* tolerance to antimicrobials, which we subsequently identified to be farnesol, a quorum-sensing molecule secreted by *C. albicans* [32]. Specifically, we demonstrated that farnesol indirectly confers *S. aureus* tolerance to vancomycin by elicitng a stress response *via* activation of the thiol-based redox system.

In addition to secreted proteins and carbohydrates one major distinguishing feature of biofilms is the presence of secreted extracellular DNA (eDNA), particularly in staphylococcal biofilms where it is crucial for biofilm structural integrity and maintenance [33–35]. In fact, the extracellular matrix of *S. aureus* biofilm is primarily composed of eDNA and the polysaccharide intercellular adhesin (PIA) [36,37]. The formation of the eDNA matrix appears to be necessary for early staphylococcal biofilm development, as early DNase treatment profoundly impairs biofilm formation [33]. In *S. aureus*, the generation of the eDNA matrix is facilitated by autolysis of a subset of initial biofilm cells, regulated by the *cidABC* and *lrgAB* operons [38–40]. Gene regulation in *S. aureus* biofilms is complex and involves the activity of several major global regulators [41,42]; however, the environment in a biofilm can be altered by the presence of co-infecting microorganisms where polymicrobial interactions can result in augmented pathogenesis. To that end, the overall goal of this study was to elucidate the impact of inter-species interactions during a biofilm-associated infection in a host. Specifically, we used an established mouse model for *in vivo* grown biofilms to demonstrate the therapeutic implications of device-associated polymicrobial infections. Further, we aimed to provide lacking insights into the transcriptional changes governing the interactions between these diverse pathogens, particularly within the context of antimicrobial resistance.

## Materials and methods

**Reagents**. MTS tetrazolium-based proliferation assay and the QuantiFluor® dsDNA System kit were purchased from Promega (Madison, WI); vancomycin hydrochloride from Hospira Inc. (Lake Forest, IL); Deoxyribonuclease I from bovine pancreas (DNAse I), Lysostaphin from *Staphylococcus staphylolyticus*, Lyticase from *Arthrobacter luteus*, Calcofluor-White and tribromoethanol (TBE) were purchased from MilliporeSigma Co. (St. Louis, MO); Concanavalin-A and vancomycin conjugated to BODYPI FL from Invitrogen (Carlsbad, CA).

**Strains and growth conditions**. The *Candida albicans* wild-type strain SC5314 [43] and the methicillin-resistant *Staphylococcus aureus* strain (USA300 LAC) were used in this study. *S. aureus* mutant derivatives of the reference WT strain defective in cell autolysis regulation (∆lrgA and ∆lrgB) were obtained from the Nebraska Transposon Mutant Library (University of Nebraska, NE). *C. albicans* was grown on yeast peptone dextrose broth (YPD) (Difco Laboratories) overnight at 30°C and *S. aureus* cultures were grown overnight in trypticase soy broth (TSB) (Difco) at 37°C. Cells were washed in Phosphate Buffered Saline (PBS) and resuspended in RPMI 1640-HEPES (Invitrogen) media to final cell density needed.

***In vitro S. aureus* vancomycin susceptibility testing in single and mixed biofilms**. Single- and mixed-species biofilms were grown in the wells of 96-well polystyrene flat-bottom plates as we previously performed [29]. *C. albicans* and *S. aureus* cell suspensions were adjusted to a final concentration of 1 × 10^6^ cells/ml in RPMI 1640-HEPES medium and 100 µl of cell suspensions were seeded in the wells individually or in combination. Plates were incubated for 90 mins at 37°C, then washed with PBS to remove non-adherent cells. Fresh medium (100 µl) was added to each well, and biofilms were allowed to form for 24 hrs at 37°C. Following incubation, wells were gently washed with PBS to remove non-sessile cells. Vancomycin (800 µg/ml) was added to wells, and plates were incubated for 24 hrs at 37°C. Following incubation, wells were sonicated to disrupt biofilms, and cell suspensions were diluted and plated on *S. aureus*-specific and *Candida*-specific chromogenic medium (CHROMagar; DRG International, Inc.) for CFU (colony forming units) enumeration. Control wells with no vancomycin were included; vancomycin concentration used was determined based on our previously published work on mixed biofilm assays [29].

**Animal studies**. All animal experiments were conducted at the AAALAAC accredited Animal Facility of the University of Maryland, Baltimore and were approved by Animal Care and Use Committee. Three-month-old female Balb/c mice were purchased from Envigo; animals received food and water *ad libitum* and were housed at a maximum of 5 mice per cage, weighed and closely monitored for any signs of distress throughout experimental periods.

**Polymicrobial catheter infection mouse model**. The previously described rodent model [44,45] was adopted, with modifications. Mice were immunosuppressed by the addition of dexamethasome (Sparhawk Laboratories; Lenexa, KS) in drinking water at final concentration of 0.4 mg/L one day prior to infection, which was maintained throughout the study. 5–8 fragments of polyurethane triple-lumen central venous catheters (1 or 0.5 cm in length) (Jorgensen Laboratories; Loveland, CO) were pre-coated overnight with fetal bovine serum (Gibco™; Waltham, MA) at 37°C. Catheters were then inoculated with single or mixed-species cell suspensions of *S. aureus* and/or *C. albicans* in RPMI 1640-HEPES at final concentrations of 1 × 10^6^ cells/ml for 90 mins at 37°C to allow for microbial adhesion to catheters. For each experimental set, *in vitro*-infected catheters were processed for assessment of microbial recovery to confirm standardized microbial adherence to catheters prior to implantation. For implantation in animals, following *in vitro* infection, fragments were rinsed with PBS and kept on ice until implanted. Mice were anesthesized by intraperitoneal injections (0.5 ml) of TBE solution (250 mg/kg body weight) and the dorsum was shaved; small incisions were aseptically made (Figure 2a-i), and a subcutaneous tunnel created allowing for insertion of 5–8 pieces of catheters (Figure 2a-ii). Incisions were sealed using 3M Vetbond™ tissue glue and lidocaine analgesic gel was applied to the incision area (Figure 2a-iii). Animals were placed in a supine position under a heating lamp and monitored until they recovered from anesthesia, and then daily for any developing clinical signs of distress. Animals were euthanized by CO_2_ inhalation followed by cervical dislocation and catheters were harvested individually.

**Scanning electron microscopy (SEM) analysis of explanted catheters for evaluation of *in vivo*-grown biofilms**. In order to visualize the biofilms formed within the lumen of the catheters, representative fragments from each group were processed for SEM analysis. Explanted catheters were rinsed with PBS then fixed in 2% paraformaldehyde, 2.5% glutaraldehyde in phosphate buffer (pH 7.4). Catheters were post-fixed with 1% osmium tetroxide in PBS for 1 hr, washed with buffer, dehydrated in a series of graded ethyl alcohol, 30%, 50%, 70%, 90%, and 3 times in 100% for 10 mins each. Specimens were critical-point dried in CO_2_, mounted on SEM pin mounts, and sputter coated with platinum-palladium (EMS 150 T ES). SEM images were acquired using a Quanta 200 scanning electron microscope (FEI Co., Hillsboro, OR), and images were processed using Adobe Photoshop software.

**Vancomycin treatment of mice with single and mixed species catheter infection**. To comparatively evaluate the therapeutic efficacy of vancomycin against *S. aureus* in animals with single or mixed catheter infections, animals were divided into a control (no therapy) group and experimental (therapy) group; within each group, 3 subgroups were included: implanted with catheters infected with *S. aureus, C. albicans* or in combination for a total of 6 groups with 5 animals in each group. Animals in the treated group were administered vancomycin twice daily by intraperitoneal injections (0.25 ml) at 55 mg/kg, and treatment was initiated on the 3^rd^ day post-catheter implantation and continued for 5 days, then animals were euthanized. Untreated control groups received sterile PBS injections, and mice with *C. albicans* mono-infected catheters were also treated with vancomycin as control. Harvested catheters were aseptically fragmented, sonicated in sterile PBS to detach biofilms and cell suspensions were diluted and plated on species-specific chromogenic media for CFU enumeration; each harvested catheter was processed individually. As pilot experiments indicated blockage of the catheters by host tissue and immune cells compromising vancomycin delivery into catheter lumen, therapy experiments were performed using longitudinally cut catheters implanted with lumen facing downwards, in direct contact with the tissue. Experiments were performed on 5 separate occasions and data combined.

**Global transcriptional analysis of single and mixed-species catheter biofilms using RNA-sequencing**. To ensure sufficient RNA yield, initial experiments were performed to determine optimum cell densities for catheter inoculation prior to implantation. Based on measurement of RNA from extracted catheters, cell densities of 1 × 10^8^ and 5 × 10^8^ cells/ml for *C. albicans* and *S. aureus*, respectively, were selected for the initial *in vitro* catheter inoculation. Implantation of catheters was performed as described above and animals were euthanized on the 3rd-day post-catheter implantation; catheters were collected in RNAlater buffer, aseptically fragmented, sonicated in RNAse free water to detach biofilms, and cells from all catheters recovered from each mouse were pooled by centrifugation. In parallel, prior to extraction, suspensions were diluted and plated for CFU enumeration to assess microbial recovery. Pooled cells were snap-frozen on dry ice and ethanol, allowed to thaw at room temperature (RT), then incubated for 30 mins at 37°C in “*digestion buffer*” containing 200 µg/ml of lysostaphin (for *S. aureus* cell wall digestion) and/or 100 U/ml of lyticase (for *C. albicans* cell wall digestion) and RNAse inhibitor in TRIS-EDTA 1x buffer. RNA was extracted in 1 ml of TRI Reagent™ Solution (Ambion, Invitrogen; Carlsbad, CA) using bead-beating for 30 mins at RT followed by purification using Direct-zol RNA Miniprep kit (Zymo Research; Tustin, CA). Eluted RNA was analyzed in a Nanodrop Lite (Thermo Scientific). Total RNA was subjected to rRNA depletion with the Illumina Ribominus Depletion kit (bacteria module) and/or the Ribominus Eukaryote Kit. All RNA-seq libraries (strand-specific, paired end) were prepared with the TruSeq RNA sample prep kit (Illumina). Seventy-five nucleotides of the sequence were determined from both ends of each cDNA fragment using the HiSeq platform (Illumina). Sequencing reads were aligned to the reference genomes (*Staphylococcus aureus* USA300 TCH1516 uid58925; *C. albicans* strain SC5314) using HISAT [46] and alignment files were used to generate read counts for each gene; statistical analysis of differential gene expression was performed using the DEseq package from Bioconductor [47]. A gene was considered differentially expressed the p-value for differential expression was less than 0.05. Two biological samples from each experimental group were analyzed. Sequencing reads were aligned to the reference genomes (*Staphylococcus aureus* USA300 TCH1516 uid58925, *C. albicans* strain SC5314)

***In vitro* evaluation of biofilm formation of the *S. aureus* lrgA and lrgB mutant strains**. Biofilm formation of *S. aureus* knock-out mutants defective in cell autolysis regulation (∆lrgA and ∆lrgB) was comparatively evaluated to the WT strain. 24 hrs biofilms were evaluated based on metabolic activity measurement using the MTS-assay as per manufacture directions (Promega; Madison, WI). Total biofilm biomass was evaluated by the crystal violet staining assay where 24 hr-grown biofilms were fixed in methanol for 20 mins, air dried and stained with crystal violet solution (0.2%) for 20 mins. The excess crystal violet was removed, plates were washed twice with PBS and cell-bound crystal violet was dissolved in 33% acetic acid. 100 µl of the extraction solution was transferred to 96-well plates and absorbance was measured at 595 nm.

***In vitro* evaluation of eDNA accumulation in single- and mixed-species biofilms**. 24 hr-preformed single and mixed-species biofilms were grown as described above using 6-well microtiter plates and eDNA was quantified as previously described with modifications [48]. Briefly, biofilm supernatant was aspirated and formed biofilms were harvested by scraping and resuspended in 1 ml PBS. Suspensions were centrifuged for 10 mins at 5000 g to separate cells and the soluble biofilm matrix and the recovered supernatant was filtered using 0.22 µm Millex syringe filters. The amount of eDNA was quantified using the QuantiFluor® dsDNA System kit (Promega, Madison, WI) as per manufacturers instructions. Fluorescence was measured at 504 nm/531 nm (Ex/Em) in triplicates per sample in flat-bottom black-sides 96-well plates (Greiner, Kremsmünster, Austria) using Biotek Cytation 5 plate reader.

**Effect of biofilm DNAse treatment on vancomycin susceptibility of lrgA and lrgB *S. aureus* mutants**. Effect of biofilm DNAse I treatment on vancomycin susceptibility was evaluated as previously described [33], with modifications. 24 hr-preformed biofilms of the WT, ∆lrgA and ∆lrgB strains were grown as described above; following removal of non-biofilm cells, DNAse I (0.625 mg/ml), vancomycin (200, 100, 50 and 25 µg/ml) or a combination of both were added to appropriate wells. Following 24 hrs incubation at 37°C, metabolic activity of biofilm cells was measured using MTS-assay. Control wells with no treatment were included; DNAse I optimal activity concentration was pre-determined in a dose–response assay (data not shown).

**Effect of biofilm DNAse treatment on *S. aureus* vancomycin susceptibility in single and mixed biofilms with *C. albicans***. 24 hr-preformed single and mixed-species biofilms were grown as described above and, after removal of non-sessile cells, DNAse I (0.625 mg/ml), vancomycin (800 µg/ml) or a combination of both were added to appropriate wells with biofilms. Following 24 hrs of incubation at 37°C, wells were sonicated, and cell suspensions were diluted and plated on species-specific chromogenic media for CFU enumeration. Control wells with no treatment were included; DNAse I optimal activity concentration was pre-determined in a dose–response assay (data not shown) and the vancomycin concentration used was based on our previously published work on mixed biofilm assays [29].

**Confocal scanning laser microscopy (CSLM) analysis of vancomycin diffusion through single and mixed-species biofilms with and without DNAse treatment**. To visualize the effect of eDNA enzymatic digestion on vancomycin diffusion through single and mixed biofilms, confocal scanning laser microscopy was performed as we previously described [29], with modifications. Single-species *S. aureus* or mixed-species *C. albicans-S. aureus* biofilms were grown on glass coverslip-bottom dishes (MatTek Co., Ashland, MA), as described above, for 24 hrs. Following incubation, biofilms were treated with DNAse I (0.625 mg/ml) or fresh RPMI (control) and fluorescent vancomycin conjugated to BODYPI FL (1 µg/ml; 488/650) was added to both control and treated biofilms, and the system was incubated for 1 hr at 37°C. *S. aureus* cells were stained with concanavalin A (ConA) conjugated to Alexa 657 (50 µg/ml; 650/668 nm) and *C. albicans* with calcofluor-white (0.05%; 433/355). Biofilms were gently washed and immediately observed in an inverted confocal laser scanning microscope (Nikon T2i). At least three random fields were visualized for each sample, and representative images of the reconstructed Z-stacks are presented.

**Data analysis**. All statistical analysis was performed using GraphPad Prism 8.0 software. All *in vitro* experiments were performed on at least 3 separate occasions and in triplicate where applicable, and averages were used to present data. Multiple groups were compared by 2-way ANOVA with Tukey’s multiple-comparison correction. The set of data obtained from WT and lrg mutants biofilm susceptibility to vancomycin alone or combined with DNAse was analyzed by 2-WAY ANOVA using the Multiple Effect analysis (Mixed Model). Student’s unpaired t-test was used to compare differences between two samples; p values of <0.05 were considered to be significant. For *in vivo* experiments, the database for statistical analysis was built up by incremental addition of experimental data. Based on our analysis, a sample size of 5 mice per experimental group will have at least 80% power to detect difference of 80%. All experiments were performed on at least three separate occasions, analyzed separately and averages used to present data. The Kruskal-Wallis one-way analysis of variance test and Dunnet’s Multiple Comparison Test were used to compare differences between multiple groups and statistical significance, respectively. P values <0.05 were considered to be significant.

## Results

**Figure 1.**
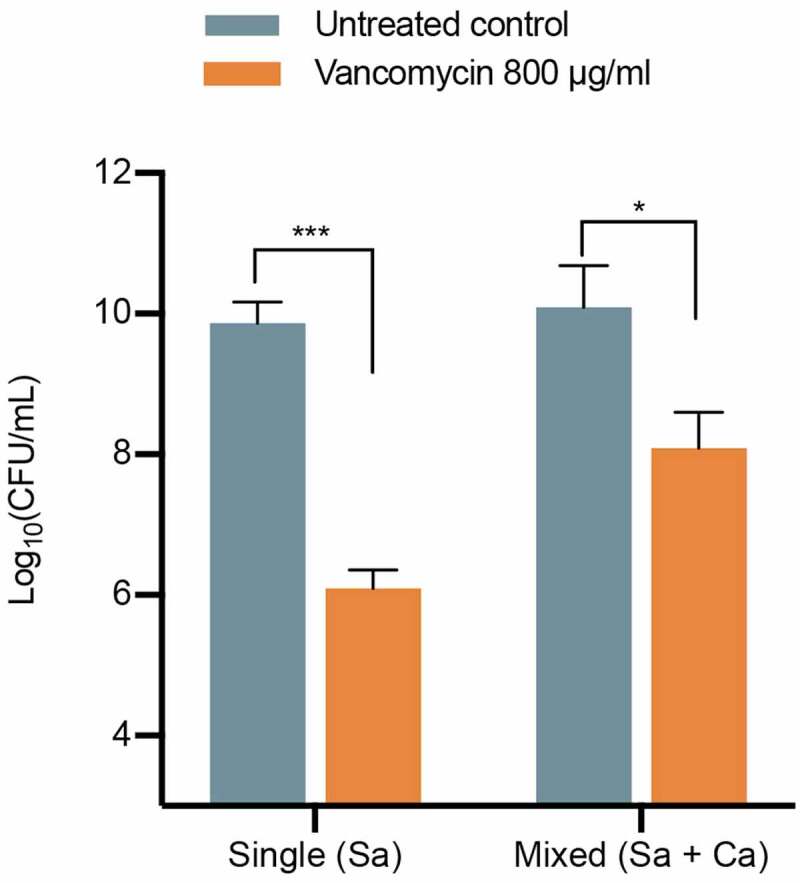
**Vancomycin susceptibility testing of single *S. aureus* and mixed *S. aureus-C. albicans* biofilms**. Single- and mixed-species biofilms were grown for 24 hrs in the wells of 96-well plates then treated with vancomycin (800 µg/ml) for 24 hrs. Following incubation, biofilms cells were harvested and plated on species-specific chromogenic medium. Based on CFU values, results demonstrated significant efficacy for vancomycin against *S. aureus* in single-species biofilms with significantly reduced efficacy against *S. aureus* in mixed-species biofilm. *, p < 0.05; ***, p < 0,001

**Mixed biofilm growth confers *S. aureus* with enhanced tolerance to vancomycin *in vitro***. C Single- and mixed-species biofilms were grown in 96-well plates using *S. aureus* USA300 and *C. albicans* SC5314 reference strains for 24 hrs and then treated with vancomycin (800 µg/ml) for 24 hrs at 37°C. Following incubation, wells were sonicated to disrupt biofilms, and cell suspensions were diluted and plated on *S. aureus*-specific and *Candida*-specific chromogenic medium (CHROMagar; DRG International, Inc.) for CFU (colony forming units) enumeration. Consistent with previous observations, significant *S. aureus* killing was seen in the vancomycin-treated single-species biofilms (****, p < 0.0001), which was significantly lower in mixed biofilms with *C. albicans* (**; p < 0.05) (Figure 1).

**Figure 2.**
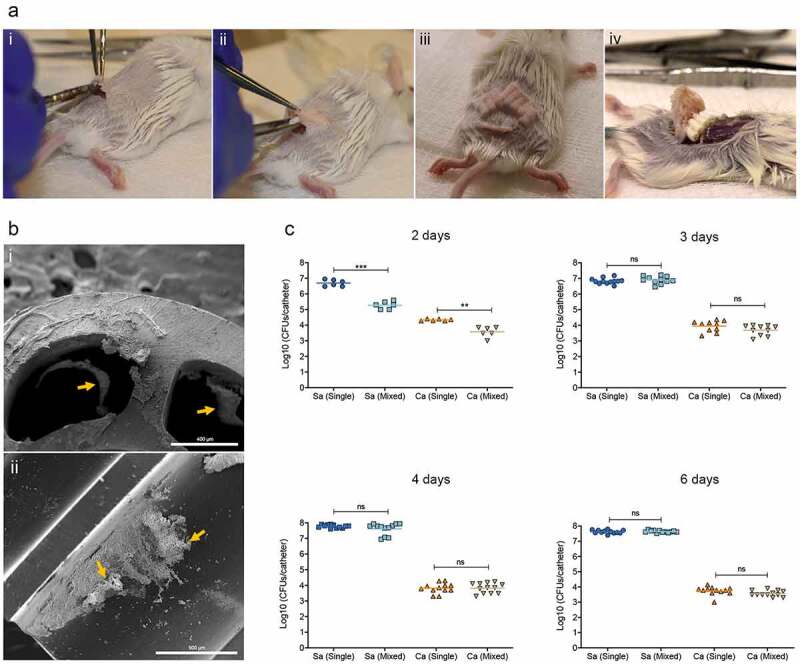
(a) **Subcutaneous catheter model for *in vivo*-grown polymicrobial biofilms**. (i) A small incision is made in a shaved area in the dorsum of the back; **(ii)** catheter fragments are inserted within a subcutaneous tunnel; **(iii)** incision is sealed with vet glue; **(iv)** catheters with formed polymicrobial biofilms are harvested from euthanized animals 2–6 days post-implantation. (b) **Scanning electron micrographs of explanted catheters with Sa-Ca mixed biofilms. (i)** Yellow arrows pointing to biofilm consisting of fungal and bacterial cells embedded in a polysaccharide matrix formed within the lumens of catheters after 8 days; **(ii)** longitudinally cut catheter fragment with biofilm. Bars = 400 µm (i) and 500 µm (ii). (c) **Time-course experiments for evaluation of microbial recovery from catheter biofilms**. Based on comparative assessment of CFUs from processed harvested catheters, 3 days post-implantation was selected as the optimal time-point for biofilm formation

**Figure 3.**
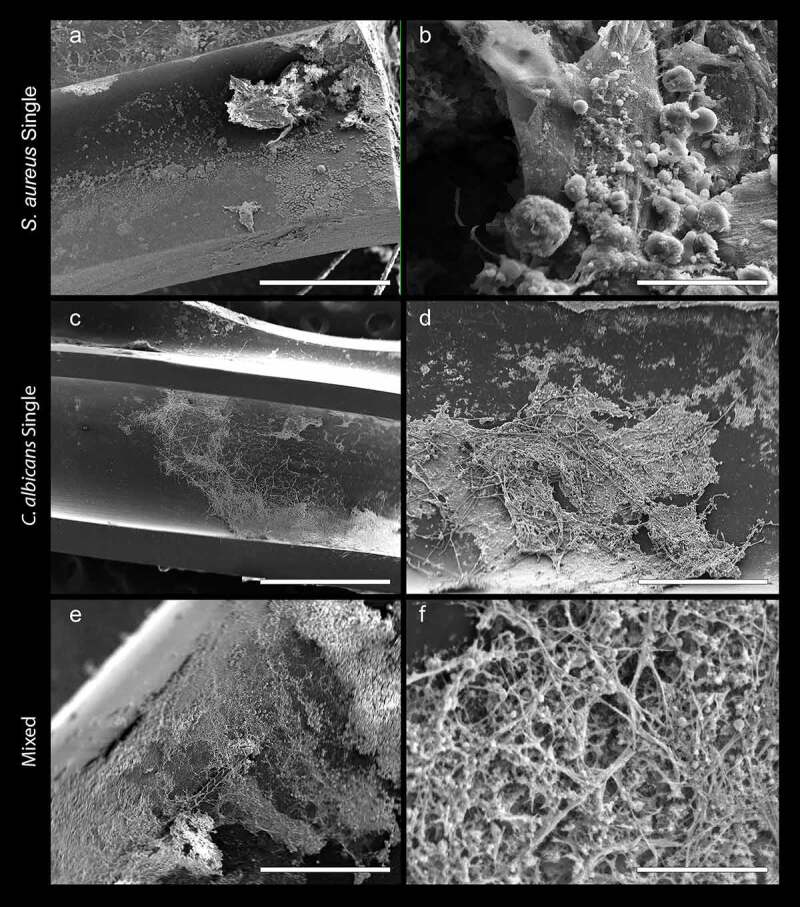
**Representative SEM images of biofilms within explanted catheters after 8 days**. Catheters infected with (a-b) *S. aureus* only; (c-d) *C. albicans* only; and (e-f) *S. aureus* and *C. albicans* (mixed biofilms). Bars: A = 400 μm; B = 20 μm; C = 1 mm; D, E = 200 μm and F = 10 μm

**Scanning electron microscopy and microbial recovery reveals formation of robust mixed biofilms within implanted catheters**. In order to determine optimal *in vivo* biofilm growth period, initially, small groups of animals with implanted catheters were euthanized two, three, four, and six days post-implantation and catheters were evaluated for microbial recovery. Based on CFU counts, significantly less *S. aureus* and *C. albicans* were recovered from mixed-species catheters after 2 days (***, p < 0.001 and **, p < 0.01; respectively), compared to from single-species (Figure 2c) and for both species CFU were equivalent in single and mixed biofilms by day 3. Based on the kinetics study, CFU recovery 3 days post-implantation was determined to be optimal time-point, after which growth of both species reached a plateau. SEM representative images of lumens of explanted catheters revealed the presence of mature mixed biofilms after 8 days (Figure 2b). Comparatively, single-species *S. aureus* biofilms had less biomass than *C. albicans-S. aureus* mixed biofilms, whereas the *C. albicans* biofilms consisting primarily of hyphae were comparable between *C. albicans* single and mixed biofilms (Figure 3).

**Figure 4.**
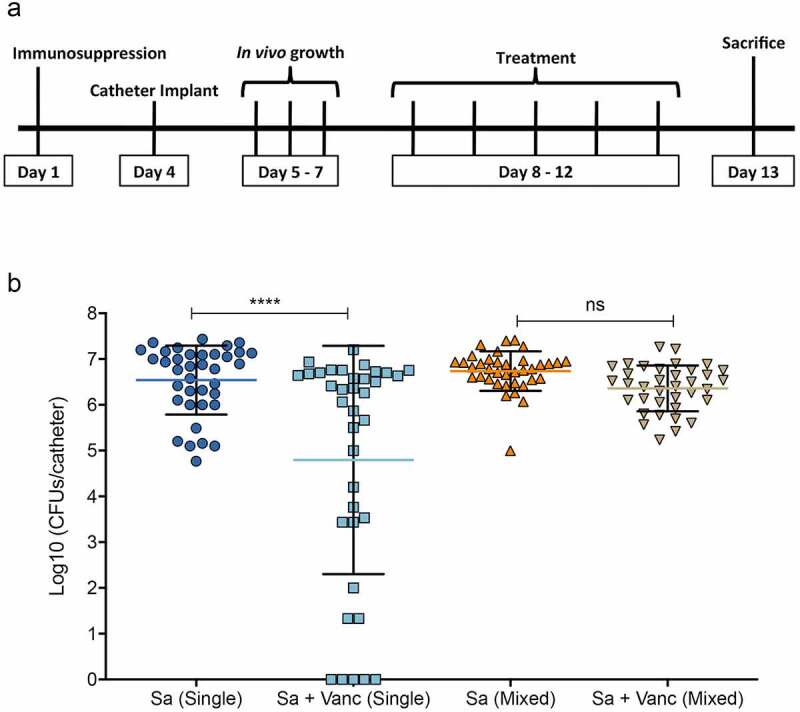
**Efficacy of vancomycin therapy in mice with catheters with single or mixed-species infection**. (a) **Timeline of infection and treatment**. Vancomycin was administered (55 mg/kg) 3 days post-catheter implantation twice daily for five days and microbial recovery from explanted catheters was evaluated. (b) Following explantation, catheters were sonicated to disrupt biofilms, and cell suspensions were diluted and plated on species-specific chromogenic media for assessment of microbial recovery. Based on CFU counts, vancomycin treatment resulted in significant decrease in *S. aureus* recovery from catheters infected only with *S. aureus*, whereas vancomycin had no effect on *S. aureus* from catheters co-infected with *C. albicans*. ****, p < 0.0001; ns, not significant

***In vivo* vancomycin therapy reduces *S. aureus* recovery from single species but not from mixed-species infected catheters**. After confirming the suitability of the model for *in vivo* biofilm development, vancomycin therapy was administered twice daily for 5 days starting 3-days post-catheter implantation (Figure 4a). Based on microbial recovery from explanted catheters, vancomycin treatment resulted in significantly less *S. aureus* recovery from catheters from animals with single-species infection (****, p < 0.0001), but had no impact on *S. aureus* recovery from animals with mixed-species catheter infections (Figure 4b). Vancomycin had no effect on *C. albicans* recovery under all conditions tested (suppl. Fig S1).

***C. albicans* modulates expression of *S. aureus* biofilm regulators in mixed-species biofilms**. Transcriptomic analysis of *S. aureus* recovered from explanted catheters revealed that 134 genes were differentially expressed between the multi-species biofilms and the *S. aureus* alone biofilms. These included 43 genes with higher expression in the multi-species biofilm and 91 genes with lower expression in the multi-species biofilm (Table 1 and S1). The most notable modulations were in *S. aureus* key-regulatory genes involved in biofilm formation, including sarU, icaB, icaC, lrgA, lrgB and nuc (Table 1). While the ica genes involved in the production of PIA (polysaccharide intercellular adhesin) were upregulated, the anti-holin like repressors of cell autolysis (lrgA and B) and the DNA nuclease (nuc) were downregulated. An overall downregulation of toxins and hemolysins was seen in *S. aureus* from mixed-biofilms including genes for gamma-hemolysins, alpha-hemolysin, leucocidin and staphylokinase; similarly, immunomodulatory and adhesion genes were also found to be down-regulated in *S. aureus* in mixed biofilms. In contrast, capsular polysaccharide biosynthesis was significantly induced by the presence of *C. albicans*. Although 114 *C. albicans* genes were found to be differentially-expressed between *C. albicans* alone and mixed-species biofilms (Table S2), Gene Ontology Analysis (GO) did not detect significant enrichment among the differentially-expressed genes. Therefore, we focused our downstream analyses on *S. aureus* genes that were significantly regulated in the mixed-species biofilms.

**Table 1.** Select *S. aureus* genes with differential expression during mixed catheter infection with *C. albicans.*

Gene	LFC ^a^	p-value	ORF description	Function
**Biofilm formation**				
sarU	2.06	0.015	accessory regulator U	positive activator of agr
icaB	1.84	0.011	intercellular adhesion protein B	PIA synthesis
icaC	1.3	0.06	intercellular adhesion	PIA synthesis
lrgA	−0.98	0.03	anti-holin like protein	repressor of autolysis
lrgB	−1.2	0.038	anti-holin like protein	repressor of autolysis
nuc	−1.32	0.002	secreted thermostable nuclease	degradation of eDNA; biofilm dispersal
**Secreted exotoxins**				
hlgC	−1.2	0.034	gamma hemolysin component C	pore forming toxin
hlgB	−1.31	0.0216	gamma hemolysin component B	pore forming toxin
hyl	−1.78	0.007	alpha-toxin (alpha-hemolysin)	pore forming toxin
lukF-PV	−1	0.045	panton-Valentine leucocidin (F)	pore forming toxin
lukS-PV	−1.6	0.0021	panton-Valentine leucocidin (S)	pore forming toxin
USA300HOU-2013	−1.81	0.0023	leucocidin subunit	pore forming toxin
USA300HOU-2011	−2.01	0.00092	leucocidin subunit	pore forming toxin
sak	−2	0.0027	staphylokinase	promotes bacterial dissemination
**Superantigens and host immune response**				
isaB	−1.152	0.026	immunodominant antigen	elicits immune response – during septicemia
set19	−1.2	0.024	superantigen like protein-toxin	activates immune response
chp	−1.51	0.002	chemotaxis inhibiting protein CHIPS	inhibits neutrophil chemotaxis
Emp	−1.3	0.021	extracellular matrix protein	adhesion to host tissue
ear	−1.5	0.012	Ear protein	superantigen
AlsS	1.28	0.025	immunodominant antigen B	immune evasion
cap5E	1.92	0.04	capsular polysaccharide biosynthesis	immune evasion
capC	1.3	0.038	capsular polysaccharide biosynthesis	immune evasion

**Figure 5.**
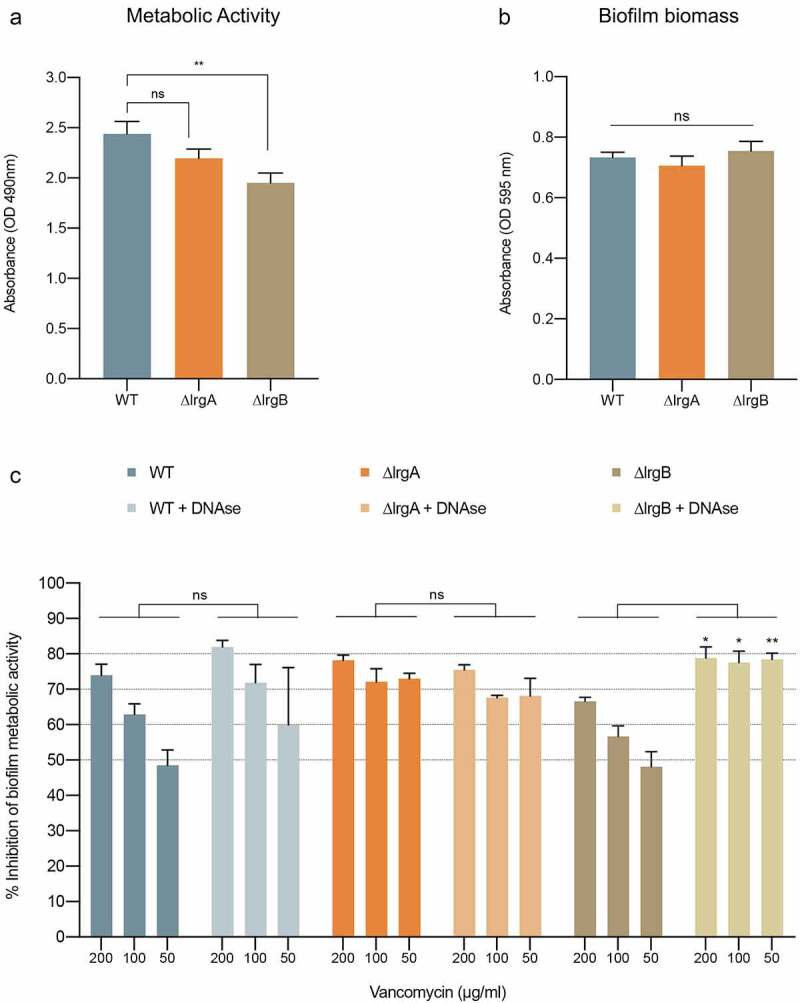
**Evaluation of biofilm formation of *S. aureus* lrg mutants defective in cell autolysis regulation (∆lrgA and ∆lrgB)**. *S. aureus* WT and derived mutants ∆lrgA and ∆lrgB biofilms were grown for 24 hrs in the wells of 96-well plates at 37°C. (a) **Biofilm metabolic activity**. Based on MTS assay on 24 hr-biofilms, ∆lrgB exhibited reduced metabolic activity (**, p < 0.01) compared to the WT strain, whereas ∆lrgA activity was comparable to that of the WT. (b) **Biofilm biomass**. Crystal violet staining indicated comparable biofilm biomass for the mutant strains and WT. (c) **Evaluation of biofilm vancomycin susceptibility with and without DNAse treatment**. 24 hr-biofilms were treated with vancomycin alone or in combination with DNAse (0.625 mg/ml) for 24 hrs and metabolic activity was quantified using the MTS-assay. Based on comparative assessment of metabolic activity of treated and untreated biofilms, ∆lrgB exhibited slightly reduced susceptibility to vancomycin compared to WT (p = 0.0147 at 200 µg/ml), which was completely reversed upon DNAse treatment. ∆lrgA was more susceptible to vancomycin than the WT at lower vancomycin concentrations concentrations (p = 0.0008 at 50 µg/ml), however no significant effect was seen upon DNAse treatment. Data set obtained in this experiment (C) was analyzed as analyzed by 2-WAY ANOVA using the Multiple Effect analysis (Mixed Model). For all experiments in this figure: *, p < 0.05; **, p < 0.001; ns, not significant

***S. aureus* lrgB mutant biofilms exhibit accumulation of eDNA in the ECM and enhanced susceptibility to vancomycin upon DNAse treatment of biofilm**. Compared to the WT strain, analysis of 24 hr-biofilms demonstrated reduced metabolic activity (**, p < 0.01; Figure 5a) for the lrgB mutant but comparable biomass (Figure 5b), and slightly reduced susceptibility to higher vancomycin concentrations (WT x ∆lrgB: p = 0.0147 using Multiple Effect analysis; Figure 5c). In contrast, the lrgA mutant exhibited similar biofilm-forming ability as the WT strain and increased susceptibility was observed for reduced vancomycin concentrations (WT x ∆lrgA: p = 0.0008 using Multiple Effect analysis; Figure 5c). DNAse treatment of biofilms had no significant impact on the susceptibility of the WT or the ∆lrgA strains to vancomycin (Figure 5c); in contrast, DNAse biofilm treatment significantly increased the susceptibility of the ∆lrgB strain to vancomycin (*, p < 0.05; **, p < 0.001; Figure 5c).

**Figure 6.**
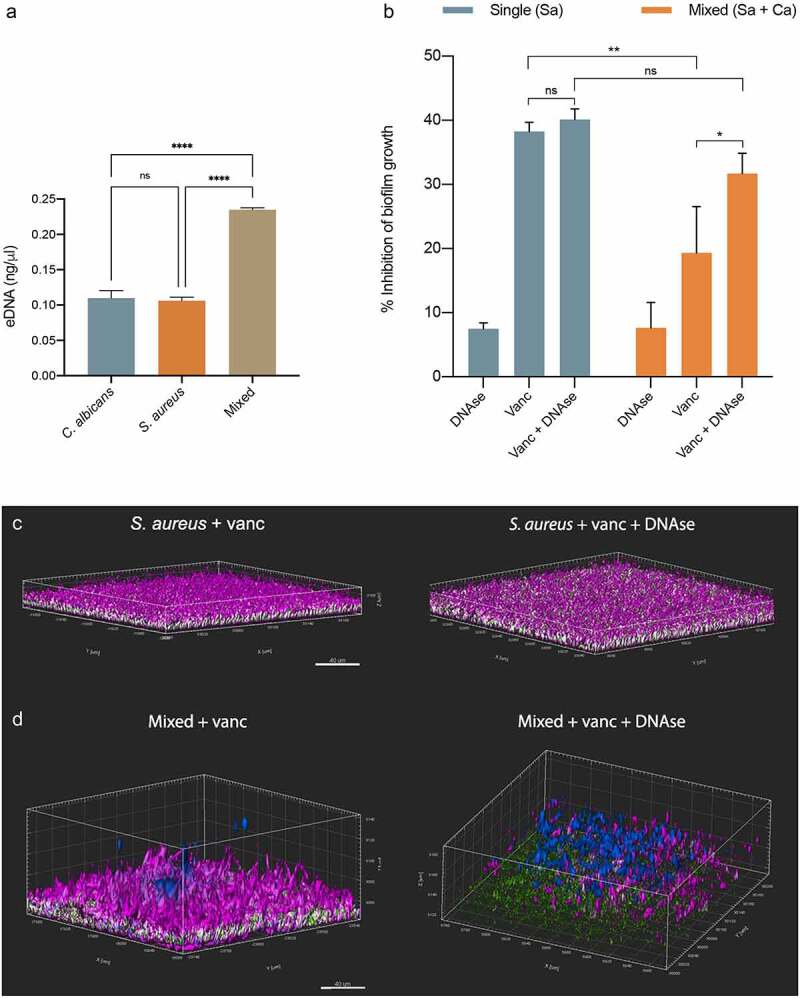
(a) **Comparartive evaluation of eDNA content in single- and mixed-species biofilms**. No significant differences in amounts of eDNA was noted in *C. albicans* and *S. aureus* single species biofilms. Significantly more eDNA was present in mixed biofilms compared to single species biofilms, individually and combined. (b-d) **Effect of DNAse treatment on biofilm structure and *S. aureus* susceptibility to vancomycin**. 24 hr-preformed single- and mixed-species biofilms were treated with vancomycin alone or in combination with DNAse (0.625 mg/m;) for 24 hrs at 37°C. (b) **Vancomycin susceptibility of DNAse-treated and untreated biofilms**. After sonication to disrupt biofilms, cell suspensions were diluted and plated on species-specific media for CFU enumeration. Results show DNAse treatment did not impact *S. aureus* susceptibility to vancomycin in single species biofilm; however, DNAse treatment significantly sensitized *S. aureus* to vancomycin in mixed-species biofilms. DNAse alone was used as negative control and showed no significant effect on biofilm viability compared to untreated controls. (c, d) **CLSM analysis of vancomycin diffusion through DNAse-treated and untreated *S. aureus* single and mixed biofilms**. Fluorescently-labeled vancomycin (green/white) was added to 24 hr pre-formed single- and mixed-species biofilms, with or without DNAse (0.625 mg/ml). *S. aureus* was stained with concanavalin A (ConA; magenta) and *C. albicans* with calcofluor-white (blue). Representative reconstructed Z-stacks are shown. (c) ***S. aureus* single-species biofilm**. DNAse treatment resulted in a more dispersed distribution of vancomycin (green/white) throughout the layers of the biofilm. (d) **Mixed-species biofilms of *C. albicans* and *S. aureus***. Unlike the effect on *S. aureus* single-species biofilm, DNAse treatment resulted in significant disruption of mixed biofilm structure, which was comcomittant with increase in vancomycin signal (green/white). Bars = 40 µm. For all experiments in this figure: ns, not significant; *, p < 0.05; **, p < 0.001; ****, p < 0.0001. Graph error bars show the standard error of the mean (SEM)

***C. albicans-S. aureus* mixed-species biofilm exhibit increased eDNA content compared to single-species biofilms**. eDNA from single species and mixed biofilm was recovered and quantitatively compared using a fluorescence-based kit. As expected, eDNA content recovered from mixed biofilms was significantly higher than that recovered from biofilms formed by *S. aureus* or *C. albicans* alone (Figure 6a).

**DNAse treatment of mixed *S. aureus-C. albicans* biofilm enhances vancomycin efficacy against *S. aureus***. To determine whether increase in eDNA contributes to the enhanced *S. aureus* tolerance to vancomycin in mixed biofilm, vancomycin susceptibility testing was performed in the presence of DNAse. Based on CFU counts, DNAse treatment of mixed biofilms significantly sensitized *S. aureus* to vancomycin (*, p < 0.05) (Figure 6b). In contrast, DNAse did not impact *S. aureus* susceptibility to vancomycin in single-species biofilm. DNAse alone had no significant effect on *S. aureus* viability compared to untreated controls.

**DNAse treatment significantly disrupts mixed-species biofilm impacting vancomycin distribution**. Confocal laser scanning microscopy (CLSM) was performed using fluorescent vancomycin to visualize the effect of DNAse treatment on vancomycin diffusion through single and mixed biofilms. *S. aureus* cells were stained with concanavalin A (ConA; magenta) and *C. albicans* with calcofluor-white (blue). Vancomycin penetration through the biofilms is seen in white and green (overlapping of green in high intensity generates the white color). Compared to untreated *S. aureus* single-species biofilm, biofilm z-stack reconstructions demonstrated that DNAse treatment resulted in a more dispersed distribution of vancomycin (green/white) throughout the layers of *S. aureus* biofilm (Figure 6c). In contrast, DNAse treatment of mixed *C. albicans-S. aureus* biofilms resulted in significant disruption in the biofilm structure, and increase in vancomycin presence (green/white) (Figure 6d).

## Discussion

Mixed fungal-bacterial infections tend to be the most complex and challenging to treat; however, the impact of these interactions on therapy remains largely understudied. *Candida* and *s*taphylococci species are the most common etiological agents isolated from device-associated nosocomial infections, and co-infection with these species poses a significant therapeutic challenge [23,24,49]. Therefore, it has become crucial to understand the mechanisms of inter-species interactions within the context of polymicrobial infections. However, a major challenge in investigating polymicrobial biofilm-associated infections is the development of suitable animal models that account for host factors, and allow for evaluation of efficacy of treatment strategies under *in vivo* conditions. Therefore, to substantiate our *in vitro* findings on enhanced *S. aureus* antimicrobial tolerance in mixed biofilms with *C. albicans* (Figure 2), we modified the established mouse subcutaneous catheter model, which proved to be suitable for sustaining polymicrobial biofilm formation, as demonstrated by SEM analysis of explanted catheters (Fig. 2, 3) [45,50]. Vancomycin therapy was implemented at clinically relevant therapeutic doses over a period of five days in mice with single-species or mixed-species infected catheters. Comparative assessment of *S. aureus* recovery from explanted catheters demonstrated inefficacy for vancomycin against *S. aureus* in mice with co-infected catheters (Figure 4). Vancomycin is one of the few antibiotics that have remained effective against methicillin-resistant *S. aureus*, and development of resistance to vancomycin is relatively rare [51]. Therefore, the demonstration of failure of vancomycin against mixed *C. albicans-S. aureus* device infection carries significant clinical implications. Notably, although single-species *S. aureus* biofilms were statistically susceptible to vancomycin treatment, the level of response was variable among the experimental sets. It is important to note however, that although the subcutaneous catheter infection model is an appropriate and reproducible model to evaluate foreign device biofilm infections and anti-biofilm strategies [45,50], one disadvantage is lack of blood flow, which can result in inefficient drug delivery to the biofilm within the catheter. In fact, an initial pilot study indicated no effect for vancomycin on *S. aureus* recovery from single-species biofilms, supporting this concern (data not shown), and therefore, to circumvent this problem, for the therapy experiments, the catheters were cut longitudinally prior to inoculation and implantation in the animals. Neverthelss, despite this limitation, the findings from using the model for *in vivo*-grown polymicrobial biofilms serve as a proof-of-principle underscoring the challenges associated with treatment of device-associated fungal-bacterial infections.

To provide insights into the mechanisms contributing to the observed therapeutic implications of mixed biofilm infections, we performed RNA-seq analysis to delineate changes in gene expression during polymicrobial infections. The most significant finding from the comparative transcriptome analysis of *S. aureus* was the down-regulation of the repressors of autolysis, lrgA and lrgB (Table 1 and Table S1), during mixed catheter infection. eDNA is essential for bacterial biofilm formation and stability, and the majority of eDNA in the biofilm matrix is a result of cell lysis, which are critical for biofilm attachment during the initial stages of development [40]. Regulatory mechanisms in *S. aureus* controlling cell death and lysis during biofilm development are mediated by the opposing functions of the cid and lrg operons encoding holin- and antiholin-like proteins, respectively, [36,39]. The lrg operon encoding LrgA and LrgB proteins are the inhibitory counterparts to cid, and a repressor of murein hydrolase activity that hydrolyzes components of the cell wall involved in autolysis. Therefore, down-regulation of lrgAB promotes the activity of the cidA-encoded holin allowing increased activity of murein hydrolases, cell lysis and eDNA production. In fact, the lrgAB mutant was shown to exhibit increased biofilm matrix-associated eDNA, consistent with its role as an inhibitor of cidA-mediated lysis [36,40,52,53]. Similarly, staphylococcal thermonuclease (nuc), which was down-regulated during mixed catheter infection, was also shown to be involved in biofilm development, and a nuc mutant was reported to form thicker biofilms containing increased levels of eDNA [40]. Therefore, cell lysis and genomic DNA release during biofilm development is regulated *via* opposing activities of the cid and lrg gene products, while staphylococcal thermonuclease functions to degrade the eDNA, possibly as a mean to promote biofilm dispersal.

*S. aureus* biofilm formation is a two-step process that requires the adhesion of bacteria to a surface followed by cell-cell adhesion or intercellular aggregation. In staphylococci, the main molecule responsible for intercellular adhesion is the polysaccharide intercellular adhesin (PIA), also called poly-N-acetylglucosamine (PNAG), which together with other polymers such as teichoic acids forms the major part of the extracellular biofilm matrix [54,55]. PIA biosynthesis is mediated by the intercellular adhesin (ica) locus, *icaADB* and *C*, with the icaB and icaC genes encoding for extracellular membrane proteins, and icaC proposed to be a putative PIA exporter [36]. Transcriptome analysis of *S. aureus* recovered from mice with mixed catheter infection demonstrated both icaB and icaC to be upregulated. Regulation of icaADBC is complex and numerous regulatory factors have been implicated, many of which are well-known global transcriptional regulatory factors like SarA and sigmaB [54]. SarA also appears to enhance biofilm formation more directly, by increasing ica expression [54,56]. Interestingly, SarU, a member of the SarA family proteins was found to be up-regulated during mixed catheter infection. Combined, the down-regulation of the lrg operon and nuc, concomitant with the upregulation of the icaADBC-encoded PIA indicate transcriptional activation of the *S. aureus* biofilm formation regulatory network, induced by the presence of *C. albicans* during mixed biofilm growth.

It is well established that biofilms are physiologically different from planktonic cells and cells in a biofilm have altered metabolic activity. In a comparative transcriptome analysis to determine which genes are up-regulated in *S. aureus* biofilm, a study by Resch *et al*. [57] found that, although the cell envelope appeared to be a very active compartment in biofilm cells as indicated by up-regulation of PIA, virulence factors such as toxins and proteases were up-regulated under planktonic growth conditions but not during biofilm growth. These findings are in line with ours, where a set of host cell-damaging proteins were found to be down-regulated during mixed species infection including gamma-hemolysins (γ-hemolysin) HlgAB and HlgCB. Interestingly, staphylococcal alpha-hemolysin (α-hemolysin) encoded by the gene *hla*, also plays a major role in the biofilm formation and appears to be primarily required for cell-to-cell interactions. In fact, a mutant allele of *hla* was shown to initially aid in colonizing a substratum. In addition to hemolysins, *S. aureus* secretes bi-component cytotoxins, Panton-Valentine leukocidins, encoded by the genes *lukSF-PV* composed of two co-transcribed open reading frames (*lukS-PV* and *lukF-PV*), which were also found to be down-regulated during mixed catheter infection [58,59]. Expression and secretion of these factors play specific roles in certain *S. aureus* life stages and diseases, and are tightly controlled by a number of regulatory systems. Most notable is the accessory gene regulator (agr), a well-characterized two-component regulatory system that plays a critical role in the up-regulation and down-regulation of protease and exotoxins, respectively, [60–62]. Interestingly, SarU, a positive activator of agr expression, is a member of the SarA family proteins; sarA is a transcriptional regulator that represses extracellular proteases and therefore, the observed elevated sarU expression during mixed catheter infection may result in additional amplification of agr signal, and explain the observed down-regulation of exotoxins in our infection model [33,63].

Interestingly, in contrast to our findings, recent studies by Todd *et al*. [64,65] aiming to elucidate observed enhanced mortality during polymicrobial *C. albicans* and *S. aureus* intra-abdominal infection, identified *C. albicans*-mediated increase in *S. aureus* secreted alpha- and delta-toxins during polymicrobial infection. Although these factors are important virulence factors in *S. aureus* crucial to its ability to cause systemic and disseminated infections, down-regulation of these factors might contribute to survival, persistence, and growth in a biofilm environment, as was seen in our biofilm infection model. Nevertheless, the contrasting regulation of *S. aureus* exotoxins mediated by *C. albicans* between the two animal models highlights the complex interactions between these two pathogens, and support the notion of niche-specific augmented pathogenesis in a co-infected host.

It is important to note that previous studies by Peters *et al*. [66] exploring the mechanism by which *C. albicans* augments *S. aureus* infection during polymicrobial intrabdominal infection, correlated lethal outcomes with exacerbation of host inflammatory responses. These findings are interesting as our transcriptome analysis of mixed-species catheter infection seemed to indicate potential dampening of host immune responses *via* down-regulation of super-antigen like proteins in *S. aureus*, such as the immunodominant surface antigen B (IsaB) which was shown to elicit immune response during septicemia [67]. These findings are in line with the overall perception that biofilms tend to be resistant to host immune defense system [68]. Interestingly, in addition to contributing to biofilm matrix, PIA molecules, which we found to be up-regulated during mixed catheter infection, also serve to shield cells from neutrophil phagocytosis, thus significantly contributing to biofilm resistance from elimination by innate host defense [26]. Most significantly however, was the noted up-regulation of *S. aureus* capsular polysaccharide biosynthesis during mixed catheter infection. The production of capsular polysaccharide by *S. aureus* has been proposed as an anti-phagocytic evasion strategy by inhibiting neutrophil phagocytosis [58]. Serotype 5 and 8 capsular polysaccharides predominate among clinical *S. aureus* isolates and animal studies have revealed that staphylococcal capsules enhance virulence by impeding phagocytosis. Furthermore, the capsule was also shown to modulate *S. aureus* adherence to surfaces *in vitro* and *in vivo*, suggesting that it also promotes bacterial colonization, and persistence in the infected host [69]

In light of the findings from the transcriptional analysis indicating enhanced *S. aureus* autolysis in mixed biofilms, and given the implication of biofilm eDNA on staphylococcal resistance to antimicrobials, as follow-up studies, we comparatively evaluated the accumulation of eDNA in single- and mixed-species biofilms. Additionally, the effect of eDNA on *S. aureus* response to vancomycin was also evaluated using DNA enzymatic digestion, and mutant strains lacking lrg operon genes. Quantification of eDNA in biofilms indicated a comparable level of eDNA produced by each species in their respective biofilms. In comparison, the total amount of eDNA in the mixed-species biofilms was significantly higher (Figure 6a). Given that both species produced eDNA and the assay is not specific, it remains speculative at this time to confirm increase in *S. aureus* eDNA specifically in mixed biofilm. It is noteworthy however that eDNA level in mixed biofilms was greater than the combined eDNA concentrations from single-species biofilms (0.2350 ± 0.0046 versus 0.216 ± 0.00989; p = 0.2592; data not shown). Nevertheless, DNAse treatment of mixed biofilms significantly sensitized *S. aureus* to vancomycin, but had little impact on *S. aureus* susceptibility in single-species biofilm (Figure 6b). The impact of DNAse on mixed biofilms was further demonstrated using confocal laser scanning microscopy where fluorescently-labeled vancomycin diffusion through DNAse treated biofilms caused significant disruption to the mixed biofilm structure, resulting in a more dispersed and accessible distribution of vancomycin (Figure 6c,D). Comparative evaluation of biofilm formation using the lrg mutant strains paradoxically demonstrated reduced metabolic activity in lrgB mutant biofilm, but no reduction in biofilm biomass, indicating decrease in cell viability due to increase in cell lysis, without impacting biofilm due to increase in eDNA content (Figure 5a-C). Theses findings were substantiated by the reduced susceptibility of the lrgB mutant to vancomycin, which was reversed upon DNAse treatment (Figure 5c). It is important to mention that the down-regulation of the lrg operon during *in vivo* grown mixed staphylococcal-*C. albicans* biofilm and inc12qqrease in biofilm eDNA content was previously reported by Pammi *et al*. [33], using a similar model. However, in that study, microarrays were used to delineate changes in *Staphylococcus epidermidis* gene expression induced by *C. albicans*. Although the authors posited that the enhanced release of eDNA due to increased autolysis would affect antibiotic tolerance, antimicrobial susceptibility assays were not performed *in vitro* or *in vivo*. However, the key role for eDNA in mediating staphylococcal tolerance to antimicrobials in single-species biofilm was clearly demonstrated by subsequent studies by Doroshenko *et al*. [70] and Brackman *et al*. [48]. In terms of therapeutic implications of the findings from our study, it is important to note an interesting study by Siala *et al*. [71] demonstrating that the antifungal caspofungin improves the activity of fluoroquinolones against *S. aureus* biofilms *in vitro* as well as in a similar subcutaneous biofilm model system as used in our study. Subsequent sequence analysis revealed a shared homology for IcaA with β-1-3-glucan synthase, the enzyme responsible for synthesis of β-1-3-glucan in *C. albicans*, and the target for caspofungin. The findings from the study did in fact show that caspofungin treatment destructured the *S. aureus* biofilm, and reduced polymerization of matrix polysaccharides increasing drug penetration into biofilms. Hence, the authors proposed that IcaA inhibitors could be useful in the treatment of *S. aureus* biofilm infections, substantiating the significance of our findings demonstrating up-regulation of the ica operon in mixed biofilms. However, as caspofungin is the drug of choice for treatment of *C. albicans*-biofilm infections, the findings from the study are of even more significance in light of ours identifying an important role for eDNA in the *C. albicans*-mediated enhanced *S. aureus* tolerance to antimicrobials. In fact, eDNA has been described as a promising new target for staphylococcal anti-biofilm treatments [35] and therefore, it is conceivable to speculate that the application of combination DNase-caspofungin treatment may prove effective against mixed *C. albicans*-staphylococci biofilm infections.

**Figure 7.**
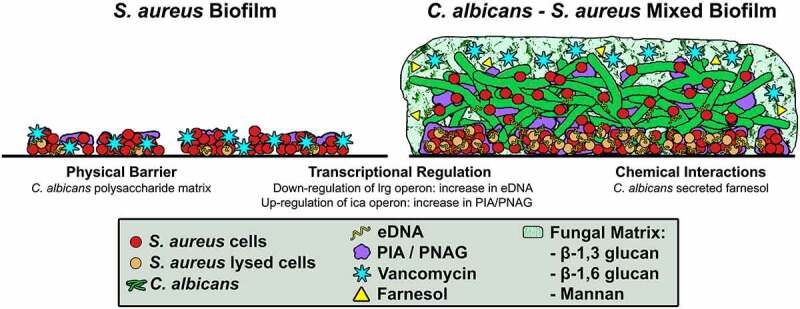
**Schematic representation of the proposed mechanisms for the *C. albicans*-mediated enhanced *S. aureus* tolerance to vancomycin**. Previously [29], we proposed a “*Barrier Model*” whereby the *C. albicans* biofilm matrix, consisting of hyphae and secreted cell wall polysaccharides (α-mannan, β-1,3-glucan and β-1,6 glucan), impedes vancomycin diffusion through the mixed-species biofilm, providing embedded *S. aureus* cells with protection against antimicrobials. However, a subsequent study [32], indicated a key role for the *C. albicans* secreted quorum sensing molecule farnesol in the enhanced *S. aureus* tolerance to vancomycin, *via* activation of a bacterial stress response. In this current study, we demonstrate that *C. albicans* induces transcriptional modulations in key *S. aureus* regulatory genes involved in biofilm formation, *via* down-regulation of the lrg operon, repressor of autolysis, and up-regulation of the ica operon, resulting in increase in eDNA and extracellular polysaccharide (PIA/PNAG) production, respectively. Therefore, in a mixed biofilm, the *C. albicans* polysaccharide matrix, combined with increase in *S. aureus* matrix components, and interactions *via* secreted chemical molecules, create a protective environment advantageous to *S. aureus.*

Combined with the findings from our previous studies [29,32], we propose that in a mixed biofilm, the *C. albicans* polysaccharide matrix, the increase in *S. aureus* matrix components, and interactions *via* secreted chemical moleucles, create a protective environment advantageous to *S. aureus* (Figure 7). Therefore, the work presented provides novel mechanistic insights into interspecies interactions in biofilms, with important clinical relevance. We expect the novel findings from this study to further our understanding of the enhanced pathogenesis and resistance of biofilm-associated polymicrobial infections, which may contribute to the development of novel therapeutic strategies targeting these complex infections.

## Supplementary Material

Supplemental Material
