# Supplementary figures and images for: Therapeutic implications of C. albicans-S. aureus mixed biofilm in a murine subcutaneous catheter model of polymicrobial infection
Source: Virulence. 2021 Mar 8;12(1):835–51. doi: 10.1080/21505594.2021.1894834 (PMC7946022; doi:10.1080/21505594.2021.1894834)

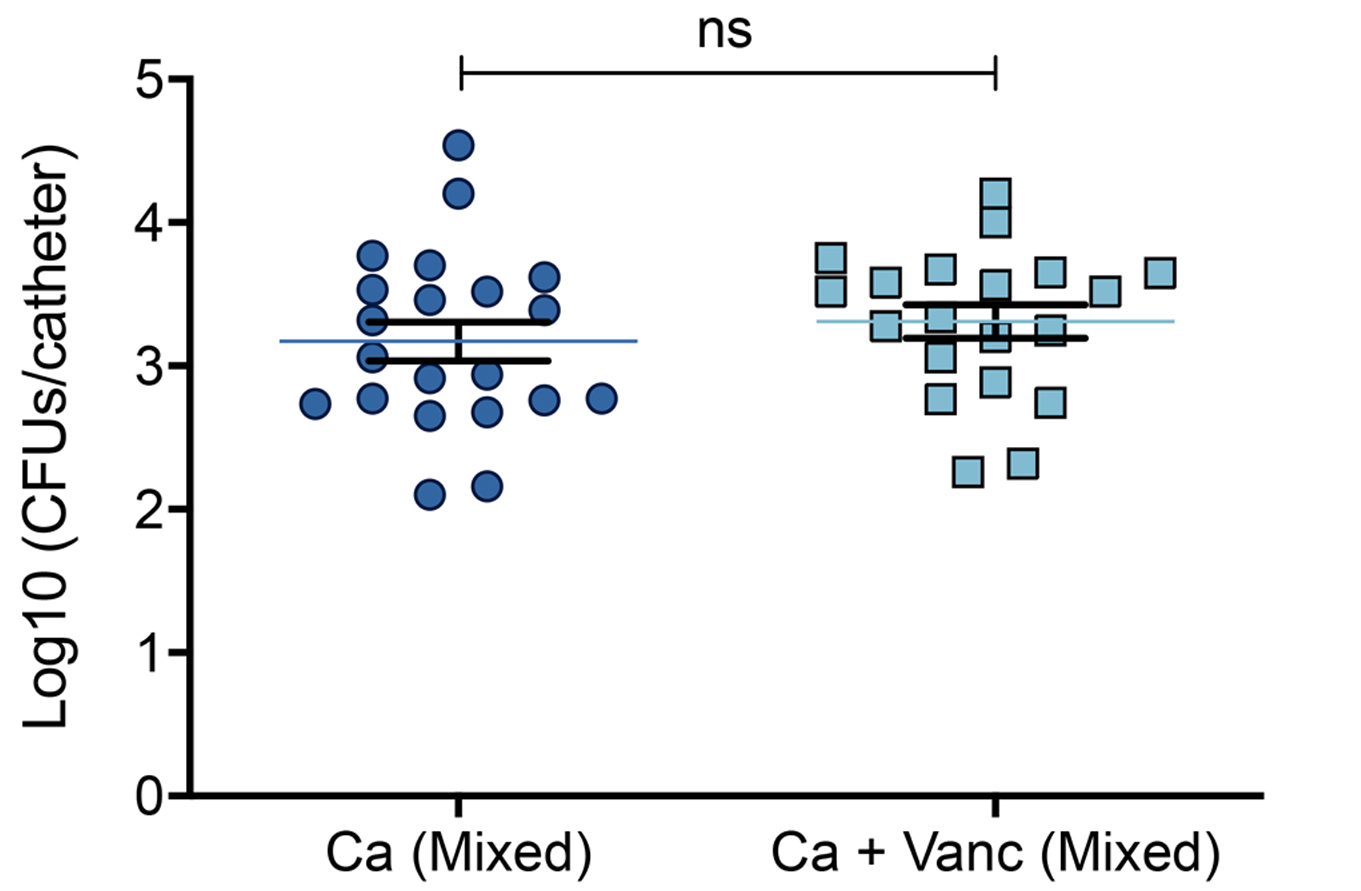

Supplement: Supplemental Material [file KVIR_A_1894834_SM4062.zip › Supplementary-FigS1.jpg]
